# Predictive Psychiatric Genetic Testing in Minors: An Exploration of the Non-Medical Benefits

**DOI:** 10.1007/s11673-017-9828-3

**Published:** 2017-12-11

**Authors:** Arianna Manzini, Danya F. Vears

**Affiliations:** 1 Neuroscience, Ethics & Society Team, Department of Psychiatry, Warneford Hospital, University of Oxford, Warneford Ln, Oxford, OX3 7JX UK; 2 Center for Biomedical Ethics and Law, Department of Public Health and Primary Care, KU Leuven, Kapucijnenvoer 35, Box 7001, 3000 Leuven, Belgium

**Keywords:** Psychiatry, Genetic testing, Children, Ethics, Autonomy

## Abstract

Predictive genetic testing for susceptibility to psychiatric conditions is likely to become part of standard practice. Because the onset of most psychiatric diseases is in late adolescence or early adulthood, testing minors could lead to early identification that may prevent or delay the development of these disorders. However, due to their complex aetiology, psychiatric genetic testing does not provide the immediate medical benefits that current guidelines require for testing minors. While several authors have argued non-medical benefits may play a crucial role in favour of predictive testing for other conditions, little research has explored such a role in psychiatric disorders. This paper outlines the potential non-medical benefits and harms of psychiatric genetic testing in minors in order to consider whether the non-medical benefits could ever make such testing appropriate. Five non-medical themes arise in the literature: psychological impacts, autonomy/self-determination, implications of the biomedical approach, use of financial and intellectual resources, and discrimination. Non-medical benefits were prominent in all of them, suggesting that psychiatric genetic testing in minors may be appropriate in some circumstances. Further research needs to empirically assess these potential non-medical benefits, incorporate minors in the debate, and include normative reflection to evaluate the very purposes and motivations of psychiatric genetic testing in minors.

## Introduction

Consider the following scenario:

*Jack is a fourteen-year-old boy with a family history of schizophrenia. His mother’s sister was diagnosed with schizophrenia at twenty years of age and his brother, now twenty-one, started showing signs of the condition one year ago. Genetic testing identified that Jack’s aunt and brother carry a copy number variation (CNV) associated with schizophrenia and Jack’s mother is a healthy carrier of the CNV. Although Jack has not shown signs of schizophrenia, he has become anxious about his potential to develop the disorder. He asks to meet with a genetic counsellor to have testing for the familial CNV.*

Predictive genetic testing (i.e. where mutations which predispose individuals to genetic conditions are tested for in currently unaffected individuals) for psychiatric conditions is not currently used in psychiatric clinical care. However, thanks to recent advances in psychiatric genetics (Ripke et al. 2014; CONVERGE 2015; Corvin and O'Donovan 2016; Sekar et al. 2016), scenarios like the one described above are likely to become part of standard practice in the not too distant future, making discussion of the ethical implications of testing timely in order to be ready to address them once they arise in practice (Appelbaum and Benston 2017). Genome-wide association studies have shown that in psychiatric disorders, the exposed attributable risk due to a CNV is quite high, meaning that for carriers, the risk of developing schizophrenia is considerable (84.1 per cent) (Gershon and Alliey-Rodriguez 2013). Some direct-to-consumer genetic companies, such as Psynomics, have previously offered psychiatric genetic testing (Couzin 2008) and the world’s first psychiatric genetic counselling clinic was founded in British Columbia in 2012 (Inglis et al. 2015), indicating that there is demand for information concerning the biological contributions of psychiatric conditions from individuals with either a personal or family history of psychiatric conditions.

Clinical use of predictive psychiatric genetic testing remains controversial due to its reduced predictive value (Lawrence and Appelbaum 2011). Although some mental illnesses have an important genetic component, in psychiatry there is no direct correlation between genotype and phenotype (Farmer, Allan, and McGuffin 2009; Porteri 2013), because the genes underlying mental disorders have complex inheritance, incomplete penetrance, and variable expression (Krystal and State 2014; Gelernter 2015). This does not exclude, though, the possibility that predictive psychiatric genetic testing will produce significant risk profiles if combined with a person’s family history and clinical data, especially once we have a better understanding of the link between biological information and phenotyping, thanks to initiatives such as the National Institute of Mental Health’s Research Domain Criteria^1^ and data analytics and pattern recognition applied to available datasets, such as the one provided by UK Biobank (McIntosh, Stewart et al. 2016).

What makes this scenario more complicated is the fact that Jack is a minor. Most national and international guidelines currently only recommend predictive genetic testing be performed in minors when it will lead to immediate medical benefits, such as treatment, prevention, or increases or decreases in surveillance. This is primarily because of concerns for the potential psychological harm from the testing itself (ESHG 2009; Botkin et al. 2015), the particular susceptibility of children and adolescents to the “social, emotional, psychosocial and educational” effects that someone else’s decision to have them tested could have on their life (Borry et al. 2008, 287), and the desire to protect the future autonomy of the child until the individual is competent to make decisions by themselves (Borry et al. 2008, Borry et al. 2009, Borry et al. 2006). Given the limited predictive power of testing, combined with the lack of consensus on the effectiveness of existing primary preventive options, predictive psychiatric genetic testing is unlikely to provide the clear and immediate medical benefits that current guidelines require for allowing testing in minors.

However, if psychiatric genetic testing is incorporated into clinical practice, it is likely to be highly relevant to children and adolescents, particularly those with a family history of mental illness, because the onset of mental disorders generally occurs in late adolescence or early adulthood. By allowing early identification, such testing has the potential to provide young people with the opportunity to prevent or delay onset of otherwise extremely debilitating disorders, either through behavioural changes or the implementation of early interventions (Appelbaum 2015).

In contrast to the guidelines, in relation to genetic testing for non-psychiatric conditions, several authors have argued that a lack of clear medical benefit is not sufficient justification for unconditionally prohibiting tests in minors as, in some situations, non-medical benefits may play a crucial role in favour of testing (Clarke 1995; Malpas 2006; Mand et al. 2012). For instance, predictive testing may improve well-being by helping at-risk minors make informed decisions about their future or integrate the knowledge about their genetic status into their self-concept (Robertson and Savulescu 2001).

To date, the ethics literature on psychiatric genetic testing has mostly concerned the potential medical harms and benefits that may be derived from performing genetic testing in minors. Although often mentioned briefly, the role that non-medical benefits could play has largely been neglected in the debates about whether testing should take place. Therefore, we conducted a review of the literature on the ethical implications of predictive psychiatric genetic testing, paying specific attention to the non-medical benefits and harms that may be derived from testing. In this paper, we outline the non-medical benefits and harms that we distilled from literature and assess whether non-medical benefits may justify predictive psychiatric genetic testing in minors under some circumstances.

## Weighing the Potential Benefits and Harms

We have identified five main non-medical categories around which the concerns regarding psychiatric genetic testing in minors are based. These are 1) the psychological impact of testing, 2) its implications for autonomy and self-determination, 3) the implications of adopting a biomedical approach to psychiatry, 4) the use of financial and intellectual resources, and 5) the discriminatory effect of testing at-risk individuals. Under each non-medical category, we present the arguments in favour and against psychiatric genetic testing in minors discussed in the literature, before considering whether the current evidence suggests that this type of testing may be appropriate under some circumstances.

### Psychological Impact

One of the most commonly referred to arguments regarding predictive psychiatric genetic testing in minors relates to the potential psychological impact of testing. The literature raises concerns that attributing a genetic basis to psychiatric conditions will increase stigma (Horstkötter, Berghmans, and de Wert 2014), which may disrupt social and family relationships. Thinking about our scenario, if Jack was found to carry the same mutation as his relatives, he may encounter disapproval, blame, or avoidance from friends, future employers, or health insurers (Nuffield Council on Bioethics 1998). This may be of greater concern if the genetic mutation in question led to susceptibility to other traits, such as antisocial behaviours, which may have implications in relation to the criminal justice system (Horstkotter et al. 2014). Identifying disease susceptibility in Jack could also change the way his parents perceive and relate to him (Erickson et al. 2014; Lee et al. 2015; Morley, Hall, and Carter 2004; Horstkötter, Berghmans, and de Wert 2014; Spriggs, Olsson, and Hall 2008). They may feel inclined to be overly protective of him (Appelbaum 2004), withdraw their affection (Florencio 2000), reduce their support of his aspirations, education, employment and relationship prospects (Corcoran, Malaspina, and Hercher 2005), or use him as the family’s scapegoat (Florencio 2000; Yeh, Morley, and Hall 2004). Given the familial nature of many psychiatric diseases, the stigma and blame associated with the condition may extend beyond the affected child, impacting on the broader family (Gershon and Alliey-Rodriguez 2013). Stigma may also affect an at-risk individual’s identity formation (Laegsgaard and Mors 2008). Identification of a mutation could undermine Jack’s sense of self and integrity (Nuffield Council on Bioethics 1998), lower his expectations about his future (Corcoran, Malaspina, and Hercher 2005), and reduce his self-esteem and self-confidence (Erickson et al. 2014; Morley, Hall, and Carter 2004).

Testing may also increase anxiety (Wilde et al. 2010; Wilde, Meiser, Mitchell, Hadzi-Pavlovic, et al. 2011a) if people overestimate their disease susceptibility or fail to comprehend complex concepts of genetic risk (Erickson et al. 2014), and a negative test result may cause false reassurance (Wilde et al. 2009). These misunderstandings are particularly problematic in children who may not have the cognitive capacities necessary to manage genetic information (Morley, Hall, and Carter 2004; Yeh, Morley, and Hall 2004). While a positive test result may be misinterpreted as a self-fulfilling prophecy, leaving no chance for risk-reducing interventions, a negative test result may be used as justification for not taking precautionary measures. In this way, tested individuals may feel discouraged from seeking help or legitimized in engaging in unhealthy behaviours, such as drug consumption or alcohol abuse (Wilde et al. 2013; Nuffield Council on Bioethics 1998). Anxiety may also trigger the development of psychiatric diseases, which could be viewed as an additional harm (Spriggs, Olsson, and Hall 2008).

However, some authors have proposed that performing psychiatric genetic testing in minors could actually reduce anxiety (Appelbaum 2004; Trippitelli et al. 1998). For example, Jack’s “experiential knowledge” based on his affected family members may lead him to perceive his risks as higher than his actual disease risk (Abel and Browner 1998; d’Agincourt-Canning 2005). Since a mutation-negative result would reduce his actual risk to that of the general population (1 per cent), testing may reduce risk-related anxiety. Anxiety may also be reduced in mutation-positive cases. On the one hand, identifying a causative CNV may create optimism in the possibility of taking action (Corcoran, Malaspina, and Hercher 2005). Of course, the capacity to act on one’s disease risk will depend on the degree of certainty associated with the result. Given the complex interaction between environmental and biological factors in psychiatric disorders, the test result will likely need to be combined with other data, such as a person’s family history and clinical data, to be informative enough to encourage agency. On the other hand, it is the limited role that genetic information alone will play in disease prediction (Appelbaum 2004) that undermines the argument that testing increases anxiety. This is because identifying a predisposing mutation would be less anxiety-provoking and have less impact on the identity of the minor compared with testing for a life-limiting condition or one that follows Mendelian inheritance, such as Huntington Disease (Newson 2009). The literature has also suggested that genetic testing may reduce the stigma associated with mental illness (Laegsgaard, Kristensen, and Mors 2009), validating them as “real” medical problems requiring intervention (Wilde et al. 2010), which may also shift responsibility for the disorder from the individual to their biological makeup (Erickson and Cho 2013).

Psychiatric testing may also have positive effects on family relationships. For example, allowing Jack to receive testing may encourage his family members to openly discuss the condition (Yeh, Morley, and Hall 2004). Parents, such as Jack’s, may also want to support their child and prevent them from suffering undue distress in the face of uncertainty. Knowing Jack carried a susceptibility CNV, might allow them to better understand their child’s behaviour if symptoms were to arise (Borgelt et al. 2014). They would also be reassured that they had not caused his condition and that they had sought help for him (Borgelt et al. 2014; Laegsgaard, Kristensen, and Mors 2009). Some parents believe testing would allow them to be better prepared to provide their at-risk children with a more protective environment, teach them resilience to counter risk factors, such as stress, and help them integrate their genetic information into their identity (Erickson et al. 2014; Wilde, Meiser, Mitchell, and Schofield 2011b). These considerations suggest parents may be motivated to have their children tested as part of what they consider to be “good parenting,” as identified in other contexts, such as caring for children who are terminally ill or desiring carrier testing in unaffected children ( Hinds et al. 2009; Vears et al. 2016, 1263). Providing their children with good care may improve the parents’ psychosocial well-being, which, in turn, may benefit the well-being of the family unit (Kattentidt-Mouravieva et al. 2014). Parents of children who received predictive testing for non-psychiatric conditions have displayed either reduced anxiety, from both mutation-positive and mutation–negative results, or felt closer to their children and better prepared to support them (Kattentidt-Mouravieva et al. 2014).

### Autonomy and Self-Determination

Another argument frequently mentioned against genetic testing in minors in both the literature and international guidelines is that children and adolescents should be given the opportunity to decide for themselves whether they want to know their genetic information when they become competent to make autonomous decisions. Although this argument is generally evoked in response to parental requests for testing, rather than when a minor requests testing, it also applies to our scenario because accommodating Jack’s wish to be tested would undermine the “future autonomy” (Nuffield Council on Bioethics 1998) of an individual who is not yet self-determinant.

Yet the same appeal to respect for autonomy has been cited in support of testing. The above argument assumes that individuals suddenly become self-determinant when they reach the age of majority (often 18 years). However, as the Convention on the Rights of the Child describes, minors show “evolving capacities” (United Nations 1989, art 5) that should limit their parents’ right to make decisions on their behalf. Using this conceptualization of childhood, “minors” do not constitute one homogeneous group (Nuffield Council on Bioethics 2015). Rather, this “developmental nature” of childhood (Nuffield Council on Bioethics 2015) means some individuals below the age of majority have the intellectual capacity and the emotional maturity to make competent decisions for themselves. Unconditionally prohibiting testing fails to recognize that some “mature minors” (Dickens and Cook 2005) are self-determinant and can participate in the decision-making process (Corcoran, Malaspina, and Hercher 2005) before they reach the legal age of majority. This is particularly relevant in cases where minors are very aware of the nature of the psychiatric condition and the potential impact on their family. Interestingly, none of the papers discussed the fact that testing in minors (especially immature minors) removes the possibility of confidentiality relating to the minor’s genetic information, given that the result would be communicated to their parents.

In a study investigating psychiatric patients’, their relatives’, and medical and psychology students’ attitudes toward psychiatric genetic testing, most participants (77.6 per cent) agreed with the idea that everyone has the right to know their genetic risk (Laegsgaard and Mors 2008). If this ethical argument applied to minors, it would follow that children and adolescents should not only be involved in the decision-making process, but they themselves could demand to have access to psychiatric genetic testing. However, as there would be no legal backing for this demand, this would be a moral rather than a legal right.

The effect of testing on parents’ autonomous choices is also debated in this context. Those against testing have argued that once psychiatric genetic testing becomes an approved practice, public interest may raise societal pressure to screen for psychiatric illness. This could undermine parental decision-making authority, either coercing parents to have their children tested or labelling them as morally irresponsible if they do not (Horstkötter, Berghmans, and de Wert 2014). However, given the complexity associated with gene–environment interactions and the potential for benefit from this as our understandings of the interactions progress, parents may seek early screening to limit their at-risk children’s exposure to environmental risk factors (Hoge and Appelbaum 2012). In this way, testing may in fact enhance parents’ autonomous choices.

### Implications of the Biomedical Approach

Some authors express concerns that taking a biomedical approach to psychiatry through the adoption of biomedical measures aiming to prevent development of psychiatric diseases could eliminate valuable personal traits from society (Nuffield Council on Bioethics 1998; Trippitelli et al. 1998), such as sensitivity, creativity (Mitchell 2000), or perseverance (Horstkotter et al. 2014). There are also concerns for a new eugenics movement (Laegsgaard and Mors 2008) which, by emphasizing the genetic causes of psychiatric illness, may degenerate into social policies similar to those seen in the twentieth century, where the socially “unfit” were eliminated to improve the “genetic stock” of society (Hoge and Appelbaum 2012, 1548). For some, a biomedical approach risks promotion of genetic determinism where the contribution of biological influences is overestimated and the role of environmental ones is underestimated (Singh and Rose 2009), which may lead to medicalization of childhood (Conrad and Schneider 1992; Horstkotter et al. 2014).

However, Horstkötter and colleagues argue that the debate surrounding genetic testing for psychiatric disorders is “bio-exceptionalist” (2014) (from the term “genetic exceptionalism” (Green and Botkin 2003)), meaning it focuses on the potential ethical drawbacks of biomedical measures that will be developed in the future and neglects that the same ethical concerns also present with psychosocial measures currently in use. In this way, the current debate overlooks the empowerment that genetic knowledge may provide to tested individuals. Such knowledge could increase their preparedness to “fight the disorder” (Laegsgaard, Kristensen, and Mors 2009), allowing them to adopt a proactive approach, by seeking information and implementing lifestyle changes to prevent disease development. If they develop signs of the condition, they may better understand themselves and their situation and seek early interventions (Erickson et al. 2014; Wilde et al. 2009; Lee et al. 2015). Empowerment may also be derived from the freedom from responsibility for developing the disease (Spriggs, Olsson, and Hall 2008; Erickson et al. 2014). Furthermore, advances in psychiatric genetics have the potential to result in a broader conception of one’s self, one’s potential, and the positive traits that can be associated with mental illnesses (Singh and Rose 2009). The challenge is to identify strategies that would encourage at-risk people’s appreciation of themselves and protect them from the debilitating effects of mental illnesses.

This perspective echoes Robertson and Savulescu’s idea of the “value of genetic knowledge,” in which they propose that current medical guidelines conceptualize benefits of testing in minors too narrowly (Robertson and Savulescu 2001, 42–43). Similarly, experiences of young people tested for conditions such as hereditary breast cancer, hereditary nonpolyposis colorectal cancer, and Huntington disease show that the knowledge gained through testing was preferential to the previous uncertainty, was perceived as empowering, and was the “missing piece” for their identity formation (Mand et al. 2013), which may also provide a counter argument to the idea that testing will have a negative impact on the young person’s sense of self, as discussed above in the Psychological Impacts section. However, the results of psychiatric genetic tests alone are likely to be less certain than those for these non-psychiatric conditions.

### Use of Financial and Intellectual Resources

The ethical debate around the use of a biomedical approach to psychiatry, as opposed to a psychosocial approach, also touches upon the way in which individual-based testing may affect society’s use of financial and intellectual resources in mental health. Supporters of testing have viewed identification of biological correlates to psychiatric conditions, and therefore validation of them as physical diseases, as an opportunity to increase government funding for mental health research (Wilde et al. 2010). However, there are concerns that increasingly describing psychiatric disorders in neurobiological terms may boost resources for research on biological interventions to the detriment of resources spent to improve psychosocial interventions, which have often proved to be very effective for patients’ recovery (Appelbaum 2017; Kong, Dunn, and Parker 2017). It should be noted, though, that the two kinds of interventions are not mutually exclusive; genetic studies can help us better understand the environmental causes of certain conditions, thus contributing to the development of more efficient psychosocial interventions (Viding 2004). This could therefore be viewed as a way to encourage “efforts to facilitate the integration of the biological and psychosocial models,” rather than as a reason to limit research on the neurobiological contributions to psychiatric disorders (Appelbaum 2017).

Some authors also hold concerns that the progressive elimination of people with mental illness will discourage researchers from conducting studies to benefit the mentally ill (Mitchell 2000). However, this would require a very accurate understanding of the role of biological mechanisms, environmental causes, and their interaction in the development of mental illness, as well as the development of very effective preventive pharmacological or psychosocial interventions. This requires a significant increase in intellectual resources devoted to address the burden of mental illness. Since our current understanding of mental illness is very distant from that objective and since mental health research is very much underfunded compared to other fields in medicine (MQ Transforming Mental Health 2015), this possibility is currently quite remote.

### Discrimination

Discrimination has implications for both the individual and society as a whole. For example, Jack’s teachers, concerned about the development of schizophrenia in their student, might reduce his coursework or encourage him to make safer choices, ultimately limiting his opportunities to learn (Corcoran, Malaspina, and Hercher 2005). There are concerns that genetic test results may create a class of “unadoptable” children (Appelbaum 2004, 346), or that Jack’s future insurers or employers could use his positive test result to deny him health coverage or employment (Wilde, Meiser, Mitchell, Hadzi-Pavlovic, et al. 2011a). Medical centres generating genetic profiles for patients to provide information about medication responses could also use data for secondary purposes (e.g. information about the serotonin transporter gene which may be associated with depression) (Appelbaum 2004; Hoge and Appelbaum 2012).

Regardless of how legitimate these concerns may be, some have criticized them as examples of “genetic exceptionalism” as previously mentioned. According to these critiques, the result of a genetic test is in no morally relevant way different from or “inherently more specific, predictive, sensitive, or private” (Holm 2007, 1196) than other health information that people are required to provide, either when they purchase health insurance coverage or by their potential employers, and therefore, does not require special status (Green and Botkin 2003). Moreover, as Raithatha and Smith (2004) show, information such as people’s sex or family history are already being used as a proxy for genetic data to calculate people’s health risks based on population studies. In other words, genetic testing is not a necessary condition for genetic discrimination, which means that people in situations similar to Jack’s could be discriminated against because of their family history of mental illness, regardless of whether testing takes place. There is already some evidence of this: in the early 2000s, three men’s employment contracts in the Hong Kong civil service were denied or terminated because they had a close relative suffering from schizophrenia (Wong and Lieh-Mak 2001). Therefore, the above arguments seem to be more of a criticism against genetic discrimination, rather than against genetic testing itself. Using this rationale, a genetic test that showed that an individual does not carry the genetic mutations that would place them at an increased risk of developing a certain condition could actually be beneficial (Appelbaum 2004).

Moreover, insurers have argued that disclosure of genetic test results for susceptibility to mental illness is necessary to increase the fairness and efficiency of the insurance system, by allowing for accurate calculations of people’s risks so that those with lower health risks are not asked to pay the same as those with higher risk (Appelbaum 2004). They also help insurers to protect themselves from the risk of “moral hazard,” where those who know themselves to be at increased risk seek health insurance while healthy people are discouraged from purchasing their coverage (Raithatha and Smith 2004; Appelbaum 2010).

Even so, it is unlikely that the availability of psychiatric genetic testing will drastically reduce the number of insurable and employable people because the logic behind insurance systems is to spread risk between many individuals (Appelbaum 2004). Indeed, in the United States, health insurers’ use of genetic information for discriminatory purposes, both in the health and employment settings, seems to be very rare (Hall and Rich 2000; Appelbaum 2010). Many countries, particularly in high-income settings, have now adopted laws, policies, and guidelines against genetic discrimination in insurance or employment contexts. For instance, in the United Kingdom, Jack’s confidentiality would be protected by the U.K. Government and Association of British Insurers’ Concordat and Moratorium on Genetics and Insurance (2014), which prohibits health insurers from forcing their customers to disclose the results of their predictive genetic tests. However, because a) most of these measures are unable to cover all possible cases of discrimination, b) proving cases of genetic discrimination is often difficult, and c) awareness of existing laws does not seem to relieve people from the fear of being discriminated against, mechanisms other than legislation alone should be used to protect individuals (Wauters and Van Hoyweghen 2016). Another possible strategy could consist of better informing the various stakeholders, including insurers and employers, about the possibilities and limits of genetic testing (Joly et al. 2017), especially for conditions like psychiatric disorders where the environment plays an important role, in order to avoid cases of prejudice about mental illness and popular misconceptions about genetic information leading to discriminatory practices (Appelbaum 2004).

At the level of society, if psychiatric testing showed a higher prevalence of susceptibility to certain psychiatric conditions in some ethnic groups, there is concern this will lead to ethnic or racial discrimination. However, if a higher prevalence is identified, this could attract intellectual resources to address mental health issues in vulnerable groups, such as ethnic minorities or those in low socioeconomic areas, which are generally neglected in research contexts (Singh and Rose 2009).

## Concluding Remarks

This is, to our knowledge, the first paper to review the non-medical arguments relating to predictive testing in minors specific to psychiatric genetics. According to our appraisal of the non-medical arguments presented in the literature, the current evidence suggests that there may be cases in which psychiatric genetic testing in minors could be considered ethically appropriate, even in the absence of medical benefit. This is not meant to dismiss the importance of the availability of medical benefits but to suggest that other factors should also be taken into account when considering whether testing should take place. In addition, as psychiatric genetics is still in its infancy (Appelbaum 2004; Hyman 2007), it should be noted that this conclusion is based on an anticipation of what psychiatric genetic testing will become, rather than its current state. Therefore, we suggest that in the future, the following points should be carefully assessed before such testing is offered in the clinic in general, let alone to minors. First, testing should be performed within the traditional healthcare setting and supported by adequate genetic counselling. Second, as with any genetic test, the analytic and clinical validity should be ensured. Third, given the complex aetiologies of psychiatric disorders, research in data science should contribute to develop risk profiles that include genetic and environmental factors, as well as family history information, in order to decrease the risk of false positives and false negatives. Fourth, the distinction between mature and immature minors in relation to their right to assent and dissent from psychiatric testing requires consideration. Finally, at least initially, consideration of performing psychiatric genetic testing should be limited to minors with a family history of that disorder.

In addition to these practical considerations, our review suggests two recommendations for future ethics research. As in the study by Mand et al. (2012), which addressed the non-medical benefits from genetic testing in minors broadly, very few arguments among those that we have identified in the literature are normative claims, such as respecting the young person’s future autonomy. Instead, most arguments are framed in ways that can be tested empirically, such as whether individuals who have been tested experience increased or reduced stigma. Future empirical research to test these empirical claims is warranted and we suggest that minors, especially those with a personal or family history of some mental illnesses, should also be involved in the debate, in order to assess the interest in, and support for, psychiatric genetic testing from those who are likely to become its main target.

However, it is unlikely that empirical studies will be sufficient to settle the dispute between those who support and those who refute testing. This is because what might be appropriate for one family may not be appropriate for another. Issues relating to predictive testing in children are also always very culturally and environmentally specific. Therefore, these decisions are, and should remain, context-dependent and focused on the needs of the individual (Lucassen and Fenwick 2012). Moreover, in order for the empirical explorations to be undertaken, predictive psychiatric genetic tests need to be in use in the clinical setting. Before this happens, we believe that there needs to be a discussion about whether we want to implement the tests in some cases in order to assess their implications or whether we consider the potential harms so problematic that any implementation would be unjustifiable. This matter will require further work where ethicists focus on a normative reflection on the very purposes and motivations of psychiatric genetic testing (e.g. should we test for the benefit of the individual or for the sake of society?) in order to evaluate whether these are worth pursing, despite the potential harms that this might derive. We believe that this fundamental issue is missing from the debate, which analyses the potential harms and benefits that may derive from testing once this is implemented in psychiatric clinical care, yet fails to explain why we should test in the first place.

## Footnotes

1
https://www.nimh.nih.gov/research-priorities/rdoc/index.shtml
